# Rapid Development of Diffuse-Type Remnant Pancreatic Cancer Following Obstructive Pancreatitis after Pancreaticoduodenectomy for Distal Bile Duct Carcinoma: A Case Report

**DOI:** 10.70352/scrj.cr.25-0715

**Published:** 2026-04-17

**Authors:** Atsushi Tomioka, Nao Kawaguchi, Yoshitaka Kurisu, Yasuhiko Ueda, Shuhei Kushiyama, Hiroki Hamamoto, Ryo Tanaka, Yoshiro Imai, Koji Komeda, Mitsuhiro Asakuma, Hideki Tomiyama, Sang-Woong Lee

**Affiliations:** 1 Department of General and Gastroenterological Surgery, Osaka Medical and Pharmaceutical University, Takatsuki, Osaka, Japan; 2 Department of Pathology, Osaka Medical and Pharmaceutical University, Takatsuki, Osaka, Japan

**Keywords:** pancreatic ductal adenocarcinoma, diffuse-type pancreatic cancer, pancreaticoduodenectomy, remnant pancreas, obstructive pancreatitis, rapid carcinogenesis, metachronous malignancy, distal bile duct carcinoma, GATA6, SMAD4

## Abstract

**INTRODUCTION:**

Distal bile duct carcinoma (DBDC) and pancreatic ductal adenocarcinoma (PDAC) are aggressive malignancies; however, their metachronous occurrence within a short postoperative timeframe is extremely rare. We present an extremely rare case of diffuse-type PDAC that rapidly developed only 16 months after curative resection of DBDC.

**CASE PRESENTATION:**

A 60-year-old woman presented with obstructive jaundice. Contrast-enhanced CT revealed circumferential wall thickening with enhancement in the intrapancreatic bile duct. Endoscopic ultrasound-guided tissue acquisition confirmed adenocarcinoma, resulting in a diagnosis of resectable DBDC. She underwent laparoscopy-assisted subtotal stomach-preserving pancreaticoduodenectomy, and the pathology demonstrated Stage IIB (pT2N1M0; UICC/AJCC 8th edition). The resection margin was negative (R0). Adjuvant chemotherapy with gemcitabine, cisplatin, and S-1 was administered for 3 months. Follow-up imaging at 16 months postoperatively revealed newly-developed and progressive main pancreatic duct (MPD) dilatation, diffuse enlargement of the remnant pancreas, and a poorly enhancing mass in the pancreatic tail. The patient also experienced epigastric and back pain, raising suspicion of obstructive pancreatitis. Endoscopic ultrasound-guided sampling again demonstrated adenocarcinoma, leading to a clinical diagnosis of *de novo* remnant pancreatic cancer. Total remnant pancreatectomy was therefore performed. Histopathological examination revealed moderately to poorly differentiated adenocarcinoma with widespread infiltration of the entire pancreas, classified as Stage III (ypT3N2M0; UICC/AJCC 8th edition). Comprehensive genomic profiling identified canonical PDAC driver alterations. These included KRAS, TP53, CDKN2A, and SMAD4, providing molecular confirmation of a *de novo* pancreatic origin. The patient died 6 months after the second surgery due to aggressive disease progression.

**CONCLUSIONS:**

This case highlights a rare, rapidly occurring metachronous combination of DBDC and highly aggressive diffuse-type pancreatic cancer. To our knowledge, this is the first case report of surgically treated remnant pancreatic cancer after DBDC with comprehensive genetic profiling. Obstructive pancreatitis secondary to pancreaticojejunostomy stricture was the most plausible underlying carcinogenic factor. Vigilant surveillance of postoperative morphological changes, particularly progressive MPD dilatation, is required to prevent delayed recognition of fatal outcomes.

## Abbreviations

95% CI
: 95% confidence interval
CA19-9
: carbohydrate antigen 19-9
CECT
: contrast-enhanced CT
CP
: chronic pancreatitis
DBDC
: distal bile duct carcinoma
EUS-TA
: endoscopic ultrasound-guided tissue acquisition
FDG
: fluorodeoxyglucose
GCS
: gemcitabine, cisplatin, and S-1
IHC
: immunohistochemistry
MPD
: main pancreatic duct
MRCP
: magnetic resonance cholangiopancreatography
MSI
: microsatellite instability
NGS
: next-generation sequencing
PD
: pancreaticoduodenectomy
PDAC
: pancreatic ductal adenocarcinoma
R0
: microscopically margin-negative resection
SIR
: standardized incidence ratio
SSPPD
: subtotal stomach-preserving pancreaticoduodenectomy
TMB
: tumor mutational burden
UICC/AJCC
: Union for International Cancer Control/American Joint Committee on Cancer

## INTRODUCTION

DBDC and PDAC share close anatomical proximity and overlapping histopathologic characteristics; however, they typically arise as distinct primary malignancies. Both are highly aggressive tumors associated with limited long-term survival,^1,2)^ and their metachronous occurrence—particularly when PDAC develops *de novo* in the remnant pancreas after PD—is extremely rare.^3)^ Diffuse-type PDAC, characterized by extensive intrapancreatic spread involving more than half of the pancreatic parenchyma, accounts for less than 5% of all PDAC cases and carries a dismal prognosis.^4–6)^ However, its pathogenesis and clinical significance remain poorly described. Herein, we report a highly unusual case of metachronous diffuse-type remnant PDAC that developed only 16 months after curative resection of DBDC, potentially related to CP induced by anastomotic obstruction after PD.

## CASE PRESENTATION

**Fig. 1:**
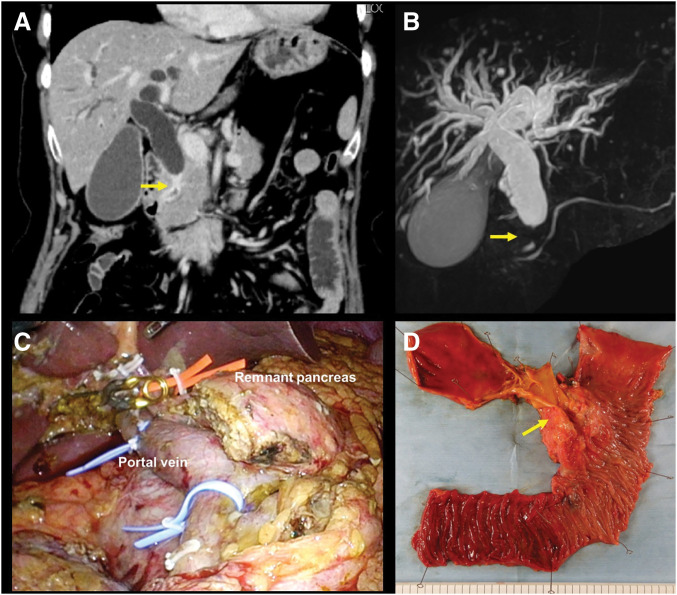
Preoperative imaging, intraoperative, and resected specimen. (**A**) Coronal view of CECT demonstrating circumferential wall thickening and intrapancreatic bile duct enhancement (yellow arrow), with upstream dilatation of both intrahepatic and extrahepatic bile ducts. (**B**) MRCP demonstrating a distal bile duct abrupt interruption and filling defect (yellow arrow). (**C**) Intraoperative findings of laparoscopy-assisted SSPPD and lymphadenectomy. (**D**) Resected specimen showing the flat infiltrative lesion along the distal bile duct wall (yellow arrow) and the intact pancreatic parenchyma. CECT, contrast-enhanced CT; MRCP, magnetic resonance cholangiopancreatography; SSPPD, subtotal stomach-preserving pancreaticoduodenectomy

**Fig. 2:**
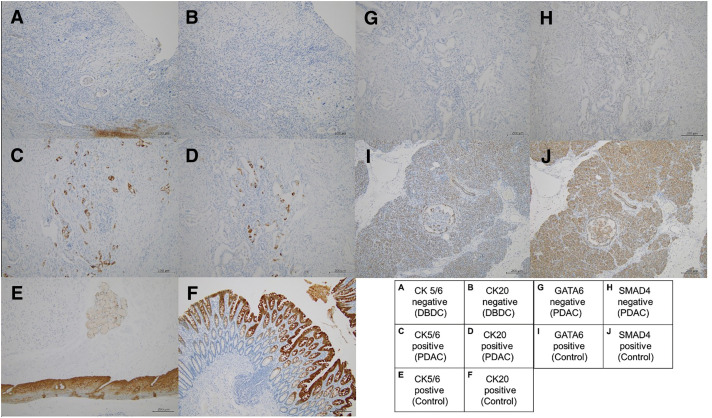
IHC of DBDC, remnant PDAC, and control tissues at ×100 magnification. Representative immunohistochemical staining for CK5/6, CK20, GATA6, and SMAD4. (**A**, **B**) DBDC demonstrating negative staining for CK5/6 (**A**) and CK20 (**B**). (**C**, **D**) Remnant PDAC illustrating positive staining for CK5/6 (**C**) and CK20 (**D**). (**G**, **H**) The same PDAC showing loss of GATA6 (**G**) and SMAD4 (**H**) expression. (**E**) Esophageal squamous epithelium serving as a positive control for CK5/6. (**F**) Colonic mucosa serving as a positive control for CK20. (**I**, **J**) Non-neoplastic pancreatic acinar cells serving as internal positive controls for GATA6 (**I**) and SMAD4 (**J**). The lower-right table summarizes immunohistochemical results for each marker in both tumors and control tissues. Original magnification: ×100. Scale bars = 200 μm. DBDC, distal bile duct carcinoma; PDAC, pancreatic ductal adenocarcinoma

A 60-year-old female patient presenting with obstructive jaundice reported a history of total hysterectomy for endometrial carcinoma at 39 years of age. She had no history of diabetes mellitus and was a lifetime nonsmoker and a social drinker. Her family history revealed a brother with colorectal cancer. CECT revealed marked circumferential wall thickening with enhancement of the intrapancreatic segment of the bile duct and prominent intrahepatic and extrahepatic bile duct dilatation (**Fig. 1A**). MRCP demonstrated a distinct filling defect in the distal bile duct (**Fig. 1B**). **Table 1** summarizes the laboratory findings before and after biliary drainage. Mild elevations in lactate dehydrogenase and gamma-glutamyl transpeptidase, an increased serum CA19-9 level of 69.8 U/mL, and post-drainage anemia with a hemoglobin level of 10.4 g/dL were observed. Tissue samples collected during EUS-TA revealed adenocarcinoma. Furthermore, along with radiologic evidence, the lesion was diagnosed as primary DBDC. No distant metastases were detected preoperatively, and the tumor was considered oncologically resectable. Therefore, the patient underwent a laparoscopy-assisted SSPPD with regional lymphadenectomy (**Fig. 1C** and **1D**). Reconstruction was performed using the modified Child method during surgery. The pancreatic texture was soft, with a narrow 3-mm MPD. A 5-Fr internal stent was placed across the pancreatojejunostomy. Postoperatively, the patient developed a clinically relevant grade B pancreatic fistula but experienced no bleeding or other major complications and was discharged on POD 27. The tumor was histologically located in the distal bile duct and exhibited a flat-type infiltrative growth pattern and was classified as a well- to moderately differentiated adenocarcinoma with focal areas of poor differentiation. The tumor invaded the fibromuscular layer of the bile duct wall without direct involvement of the pancreas or surrounding organs, and the depth of invasion was 9 mm (pT2). Lymphatic and venous invasions were present (Ly1, V1), whereas perineural invasion was absent (Pn0). Of 29 regional lymph nodes, 2 were positive for metastasis (pN1), and no distant metastasis was identified (cM0). All surgical margins were microscopically negative (R0). According to the UICC/AJCC 8th edition, the final pathological stage was determined as Stage IIB (pT2N1M0). IHC revealed CK20 (−) and CK5/6 (−) (**Fig. 2**). In the present case, adjuvant chemotherapy with GCS was initiated in the early postoperative period because CECT follow-up revealed a small hepatic lesion for which liver metastasis could not be completely excluded. During subsequent follow-up, the hepatic finding became radiologically inconsistent with metastasis, and chemotherapy was discontinued after 3 months in accordance with the patient’s preference. The imaging findings related to this clinical course are shown in **Supplementary Figure 1**. Four months postoperatively, follow-up CECT confirmed that the pancreatic duct stent remained in place. At 8 months, the internal stent had migrated spontaneously, as expected. A CECT scan at 10 months revealed no abnormalities in the remnant pancreas.

**Table 1:** Laboratory data obtained before and after biliary drainage

Laboratory test	Before biliary drainage	After biliary drainage	Unit
WBC		4780	/μL
RBC		3.68 × 10^6^	/μL
Hb		10.4	g/dL
Ht		32.1	%
Plt		20.6 × 10^4^	/μL
BUN		18	mg/dL
Cr		0.61	mg/dL
TP		5.8	g/dL
Alb		4	g/dL
T-Bil	7.1	1.2	mg/dL
AST	292	28	U/L
ALT	404	29	U/L
LDH	371	196	U/L
ALP	699	148	U/L
γ-GTP	1026	83	U/L
AMY		150	U/L
CRP		0.15	mg/dL
HbA1c		5.2	%
CA19-9	9.8	69.8	U/mL
CEA	1.4	1.8	ng/mL

Alb, albumin; ALP, alkaline phosphatase; ALT, alanine aminotransferase; AMY, amylase; AST, aspartate aminotransferase; BUN, blood urea nitrogen; CA19-9, carbohydrate antigen 19-9; CEA, carcinoembryonic antigen; Cr, creatinine; CRP, C-reactive protein; Hb, hemoglobin; HbA1c, hemoglobin A1c; Ht, hematocrit; LDH, lactate dehydrogenase; Plt, platelets; RBC, red blood cells; T-Bil, total bilirubin; TP, total protein; WBC, white blood cells; γ-GTP, gamma-glutamyl transpeptidase

**Reference ranges:** Within institutional standard limits unless otherwise specified.

However, at 15 months postoperatively, the patient presented to the emergency outpatient clinic with epigastric and back pain that had persisted for more than 1 week. Abdominal plain CT demonstrated dilatation of the MPD. No peripancreatic inflammatory changes suggestive of acute pancreatitis were observed, and laboratory tests, including pancreatic enzymes and inflammatory markers, were within normal limits. As the pain was tolerable, she was treated conservatively with analgesics and discharged home.

**Fig. 3:**
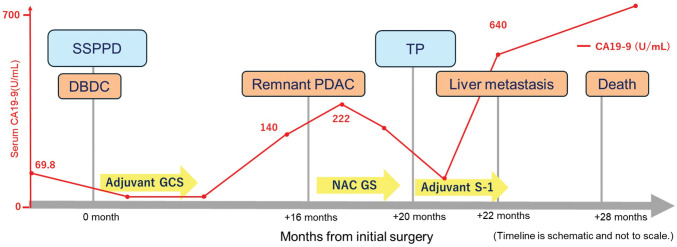
Clinical course and serum CA19-9 trend. The timeline is expressed in months from the initial surgery. Adjuvant GCS was administered after the first DBDC. After 16 months, a *de novo* remnant pancreatic cancer developed, treated with neoadjuvant chemotherapy and subsequent TP. The serum CA19-9 level rapidly increased before both PDAC diagnosis and death. CA19-9, carbohydrate antigen 19-9; DBDC, distal bile duct carcinoma; GCS, gemcitabine, cisplatin, and S-1; PDAC, pancreatic ductal adenocarcinoma; TP, total pancreatectomy

**Fig. 4:**
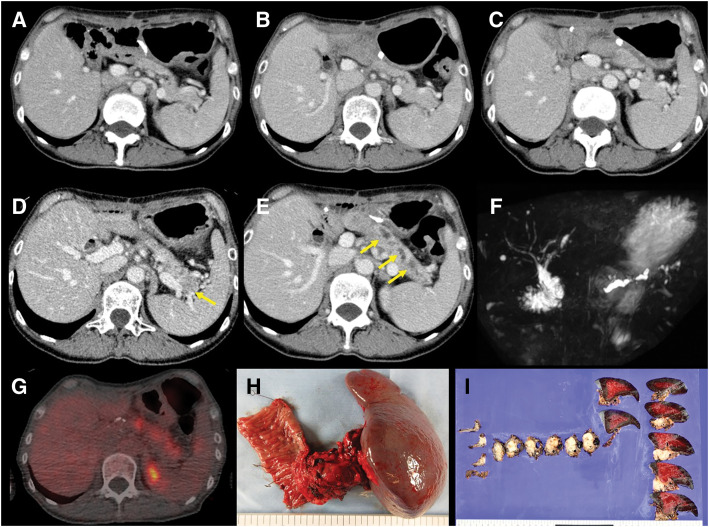
Serial CECT and MRCP images at 4, 8, 10, and 16 months post-surgery. (**A**) Four months postoperatively, no abnormalities were observed in the remnant pancreas. The internal stent remained in place. (**B**) At 8 months, no abnormalities were observed in the remnant pancreas. The internal stent had spontaneously migrated as expected. (**C**) At 10 months, no abnormalities were observed in the remnant pancreas. (**D**) At 16 months, CECT demonstrated a hypodense area in the pancreatic tail (yellow arrow). (**E**) At 16 months, CECT demonstrated diffuse dilation of the MPD and a hypodense area in the pancreatic tail (yellow arrows). (**F**) MRCP at 16 months revealed diffuse dilatation of the MPD throughout the remnant pancreas. (**G**) FDG-PET/CT at 16 months demonstrated diffuse uptake of FDG in the remnant pancreas. (**H**, **I**) Resected specimen demonstrating diffuse infiltration of tumor throughout the pancreas. CECT, contrast-enhanced CT; FDG-PET/CT, 18F-fluorodeoxyglucose PET/CT; MRCP, magnetic resonance cholangiopancreatography

At 16 months postoperatively, CECT demonstrated progressive MPD dilatation and a newly detected low-attenuation lesion in the pancreatic tail, accompanied by an increase in serum CA19-9 to 140 U/mL (**Figs. 3** and **4**). PET/CT demonstrated diffuse and intense FDG uptake throughout the remnant pancreas (**Fig. 4G**). EUS-TA again demonstrated adenocarcinoma, leading to a clinical diagnosis of *de novo* remnant pancreatic cancer. After neoadjuvant chemotherapy with 2 cycles of gemcitabine plus S-1 regimen, completion total remnant pancreatectomy and splenectomy were performed. Intraoperative findings revealed a diffusely firm and fibrotic pancreas, as well as markedly hardened surrounding tissues due to chronic inflammation. No apparent peritoneal dissemination or liver metastasis was observed intraoperatively. The pathological diagnosis was moderately- to well-differentiated tubular adenocarcinoma. The tumor was predominantly nodular but also demonstrated infiltrative growth at the peripheral tumor margin and showed diffuse infiltration of the entire pancreas. The tumor measured 65 mm and invaded the serosa, duodenal wall, and portal vein (ypT3). Lymphatic (Ly1), venous (V1), and perineural invasion were observed (Pn1). Ten regional lymph nodes were positive for metastasis (ypN2). The surgical margins were negative (R0). According to the UICC/AJCC 8th edition, these findings corresponded to Stage III (ypT3N2M0). IHC revealed CK20 (+), CK5/6 (+), GATA6 (−), and SMAD4 (−) (**Fig. 2**). These findings indicated a basal-like phenotypic subtype of PDAC.^7)^ Comprehensive genomic profiling was performed on formalin-fixed, paraffin-embedded tumor tissue from the remnant pancreatectomy specimen using a hybrid-capture, NGS assay (FoundationOne CDx, Foundation Medicine, Cambridge, MA, USA), following the manufacturer’s validated analytic protocols. This assay interrogates 324 cancer-related genes for base substitutions, short insertions/deletions, copy-number alterations, and selected gene rearrangements, and evaluates key genomic biomarkers, including MSI and TMB, when feasible. The assay revealed pathogenic or likely pathogenic alterations in *KRAS (G12V)*, *TP53* (splice-site c.376-1G > A), *CDKN2A* (A21fs*5), and *SMAD4* (A118V)—all 4 canonical PDAC driver genes collectively known as the “Big 4.” No reportable alterations were identified in *BRCA1/2*. MSI and TMB were reported as “Cannot Be Determined,” probably due to low tumor purity. Thus, no actionable genomic alterations with therapeutic implications were identified. Subsequently, she developed rapidly progressive multiple liver metastases 2 months after the second surgery and died 6 months later due to poor tolerance to chemotherapy and uncontrolled disease progression. **Figure 3** illustrates the clinical course of the patient.

## DISCUSSION

DBDC and PDAC are both aggressive malignancies; however, their sequential development within a short duration is extremely rare. We present a case of DBDC followed by diffuse-type PDAC within just 16 months, demonstrating loss of SMAD4 and GATA6 expression, indicating highly aggressive biological behavior and poor prognosis.

The key diagnostic challenge was whether the pancreatic lesion represented a recurrence of the previous bile duct carcinoma or a newly developed *de novo* PDAC. The initial tumor demonstrated typical radiological and pathological features of DBDC and no pancreatic invasion, thereby making a pancreatic origin unlikely. Several findings strongly supported the second lesion as a newly developed PDAC: (1) diffuse intrapancreatic spread without continuity from the bile duct stump; (2) distinct immunophenotypic differences, particularly the differential expression of CK5/6 and CK20 between the 2 tumors; and (3) the presence of all 4 canonical PDAC driver mutations (*KRAS*, *TP53*, *CDKN2A*, and *SMAD4*).

Among these, the third finding was particularly compelling, as the coexistence of these “Big 4” driver mutations represents a mutational signature virtually pathognomonic for PDAC.^8)^ Altogether, these findings confirmed the pancreatic lesion as a newly developed remnant pancreatic cancer after curative resection of the DBDC.

Previous reports describing the development of remnant pancreatic cancer after PD for DBDC are limited, and only sporadic cases have been documented (**Table 2**). The reported interval to the onset of remnant pancreatic cancer varies widely, ranging from approximately 18 months to more than a decade. In the present case, the tumor was first detected 16 months after the initial surgery, placing it among the earliest-onset cases reported to date.

**Table 2:** Literature review of remnant pancreatic cancer following PD for DBDC

Year	Author	Age	Sex	Indication for PD	Interval to remnant pancreatic cancer (mo)	Operation	Recurrence	Prognosis (mo)	Alive/Death
2010	Carboni et al.^12)^	48	F	DBDC	84	TRP	No	30	Alive
2014	Sunagawa et al.^13)^	72	F	DBDC	18	TRP	N/A	N/A	
2017	Ando et al.^14)^	60	F	DBDC	19	TRP	Yes	16	Alive
2018	Wakasa et al.^15)^	82	M	DBDC	46	PR	No	6	Alive
2020	Takagi et al.^16)^	75	M	DBDC	56	TRP	Yes	24	Alive
2021	Yamane et al.^17)^	67	M	DBDC	66	TRP	No	66	Alive
2022	Kim^18)^	75	F	DBDC	216	TRP	No	13	Alive
2026	Tomioka et al.	60	F	DBDC	16	TRP	Yes	6	Death

DBDC, distal bile duct carcinoma; F, female; M, male; mo, months; N/A, not available; PD, pancreaticoduodenectomy; PR, partial resection; TRP, total remnant pancreatectomy

In many of the published cases, total remnant pancreatectomy was performed; however, this reflects the standard surgical approach for tumors arising in the remnant pancreas and does not necessarily indicate advanced disease. In our case as well, total remnant pancreatectomy was selected. Notably, the primary tumor focus was located in the pancreatic tail, with diffuse infiltration extending throughout the pancreas, particularly along the subepithelial region of the MPD, which represents a characteristic feature of this case.

With regard to prognosis, follow-up periods in previously reported cases have generally been limited, and clear disease-related deaths have not been documented, with only a small number of recurrences described. By contrast, the present patient died just 6 months after total remnant pancreatectomy, demonstrating an extremely rapid and aggressive clinical course compared with previously reported cases. Thus, this case may represent not merely another example of remnant pancreatic cancer, but a biologically highly aggressive disease entity.

Taken together, the key characteristics of this case are: (1) a diffusely infiltrative growth pattern, (2) an exceptionally poor prognosis compared with previously reported cases, and (3) development within a relatively short postoperative interval. In the following sections, we discuss the pathological and genetic features and potential mechanisms underlying this unusual clinical course in the context of diffuse-type pancreatic cancer.

The so-called diffuse-type PDAC is a rare and aggressive variant of PDAC, accounting for less than 5% of all cases. It is characterized by extensive intrapancreatic spread that involves more than 50% of the pancreatic parenchyma. However, its molecular background and prognostic implications remain poorly understood.^4,5,9)^

The tumor was GATA6-negative and CK5/6-positive, consistent with the basal-like subtype proposed by Richard A. Moffitt et al., which is associated with poor prognosis and resistance to chemotherapy.^10)^ Furthermore, SMAD4 loss, reported in approximately half of PDACs, correlates with increased metastatic potential and shorter overall survival.^11)^ In our case, the aggressive clinical course may reflect the combined biological effect of SMAD4 loss and GATA6 negativity. However, their specific roles in diffuse-type PDAC have not been systematically investigated.

Metachronous occurrence of bile duct and pancreatic cancers is rare.^3,12–18)^ Furthermore, the diffuse-type presentation is even more exceptional. Considering the rapid sequence of tumor development, the possibility of an underlying germline mutation was first considered.

Known germline alterations that increase the risk of both pancreatic and bile duct cancers include those in the homologous recombination repair pathway (*BRCA1/2*, *PALB2*, and *ATM*), the mismatch repair pathway (*MLH1*, *MSH2*, *MSH6*, *PMS2*, and *EPCAM*), and *STK11* associated with Peutz–Jeghers syndrome.^19–21)^

However, the current NGS analysis detected none of these mutations. Pancreatobiliary maljunction and chronic inflammation have also been suggested as shared risk factors; in the present case, pancreatobiliary maljunction was not identified.

Therefore, a plausible mechanism for the development of remnant pancreatic cancer in the present case is CP secondary to pancreatic duct obstruction. A systematic review and meta-analysis reported that the SIR of PDAC in patients with CP is 22.61 (95% CI: 14.42–35.44). Notably, this elevated risk persisted even after excluding patients diagnosed with PDAC within 2 years of CP diagnosis to minimize detection bias (SIR 21.77, 95% CI: 14.43–32.72) 24).

On serial CT imaging, the remnant pancreas and the MPD remained unremarkable up to 10 months after PD (**Fig. 4A**–**4C**). By 16 months postoperatively, however, a newly developed hypodense lesion was identified in the pancreatic tail, accompanied by diffuse, irregular (“beaded”) dilatation of the MPD (**Fig. 4D** and **4E**). Concurrently, at 15 months, the patient developed persistent epigastric and back pain with progressive ductal dilatation, suggestive of obstructive pancreatitis due to pancreatojejunostomy stricture (**Table 3** summarizes the chronological changes). On CECT and MRCP, the MPD showed an irregular beaded configuration with alternating dilatation and narrowing, rather than the uniform dilatation typically seen with simple downstream obstruction. Although a beaded appearance can be observed in CP, it is generally regarded as a late morphological feature reflecting longstanding inflammatory change. In our case, pathological examination confirmed that the main tumor was located in the pancreatic tail; however, scattered tumor foci were also identified beneath the epithelium of the MPD throughout the remnant pancreas. Therefore, in addition to obstruction-related ductal dilatation associated with a pancreatojejunostomy stricture, focal ductal narrowing due to tumor infiltration likely coexisted. We speculate that the combination of obstruction-induced dilatation and segmental tumor-related strictures contributed to the beaded morphology observed on imaging.

**Table 3:** Chronological changes in the remnant pancreas after PD

Months after PD	4	8	10	15	16
**Internal stent**	Present	Present	Migrated	–	–
**MPD dilatation**	No	No	No	Yes	Yes
**Symptoms**	No	No	No	Epigastric/back pain	No
				> 1 week	
**CA19-9 (U/mL)**	8.2	7	9.2	N/A	140
**Secondary tumor**	No	No	No	Not detected	Present

Serial changes in internal stent status, MPD dilatation, symptoms, and CA19-9 levels.

CA19-9, carbohydrate antigen 19-9; MPD, main pancreatic duct; N/A, not available; PD, pancreaticoduodenectomy

Importantly, possible causes of ductal dilatation in this setting include both benign anastomotic stricture and tumor-related narrowing. Histopathological evaluation of the pancreatojejunostomy site in the completion pancreatectomy specimen showed tumor extension close to the anastomosis; thus, it was difficult to determine whether the obstruction initially arose from benign fibrotic scarring or from malignant infiltration. The possibility that a rapidly progressing tumor in the pancreatic tail secondarily obstructed the anastomosis cannot be completely excluded. Nevertheless, 3 key chronological and clinical observations support a primary contribution of postoperative anastomotic stricture: the primary tumor was pathologically centered in the pancreatic tail, the tail lesion was detected after the pancreatic duct stent had migrated/dislodged, and the patient had a history of Grade B postoperative pancreatic fistula—an environment in which benign anastomotic stricture may develop following stent migration. Therefore, while the proposed mechanism remains a hypothesis based on the clinical course, these findings overall support our hypothesis that postoperative anastomotic stricture led to pancreatic duct dilatation and chronic obstructive pancreatitis, contributing to carcinogenesis in the distal remnant pancreas.

In the present case, the interval between the suspected onset of ductal obstruction—at least after migration of the internal stent 10 months after PD—and the development of pancreatic cancer was estimated to be less than 6 months. This interval is exceptionally short, even considering the markedly increased risk of PDAC in patients with CP. Nevertheless, the occurrence of diffuse-type PDAC in the remnant pancreas within such a short interval after PD underscores the need for vigilant postoperative monitoring in similar settings.

## CONCLUSIONS

This case represents an exceptionally rare and biologically aggressive metachronous malignancy, emphasizing the need for further clinical and molecular investigations to clarify the mechanisms underlying diffuse-type pancreatic cancer. The abrupt development and progression of diffuse-type pancreatic cancer after PD may have been driven by a combination of relatively abrupt pancreatic duct obstruction and adverse molecular alterations, including loss of GATA6 and SMAD4. To our knowledge, this is the first case report of surgically treated remnant pancreatic cancer after DBDC with comprehensive genetic profiling. Postoperative morphological changes in the remnant pancreas—especially progressive dilation of the MPD—should be closely monitored, as such findings may herald early neoplastic transformation.

## SUPPLEMENTARY MATERIALS

Supplementary Figure 1 Chronological imaging course of transient hepatic lesions after surgery. (A-1,2) CECT performed 2 months postoperatively showed 4 small hepatic lesions (yellow arrow). (B-1,2) At 4 months postoperatively (1 month after initiation of the GCS regimen), all 4 lesions had disappeared on contrast-enhanced CT. (C-1,2) At 11 months postoperatively (6 months after cessation of GCS), no recurrence of these lesions was observed on EOB-MRI.

## DECLARATIONS

### Availability of data and materials

The data that support the findings of this study are available from the corresponding author upon reasonable request.
