# Supplementary material for: Rapid Development of Diffuse-Type Remnant Pancreatic Cancer Following Obstructive Pancreatitis after Pancreaticoduodenectomy for Distal Bile Duct Carcinoma: A Case Report
Source: Surg Case Rep. 2026 Apr 17;12(1):25-0715. doi: 10.70352/scrj.cr.25-0715 (PMC13092357; doi:10.70352/scrj.cr.25-0715)
Supplement: Supplementary Figure 1 — Chronological imaging course of transient hepatic lesions after surgery. (A-1,2) CECT performed 2 months postoperatively showed 4 small hepatic lesions (yellow arrow). (B-1,2) At 4 months postoperatively (1 month after initiation of the GCS regimen), all 4 lesions had disappeared on contrast-enhanced CT. (C-1,2) At 11 months postoperatively (6 months after cessation of GCS), no recurrence of these lesions was observed on EOB-MRI. [file scr-12-01-25-0715-s001.pdf]

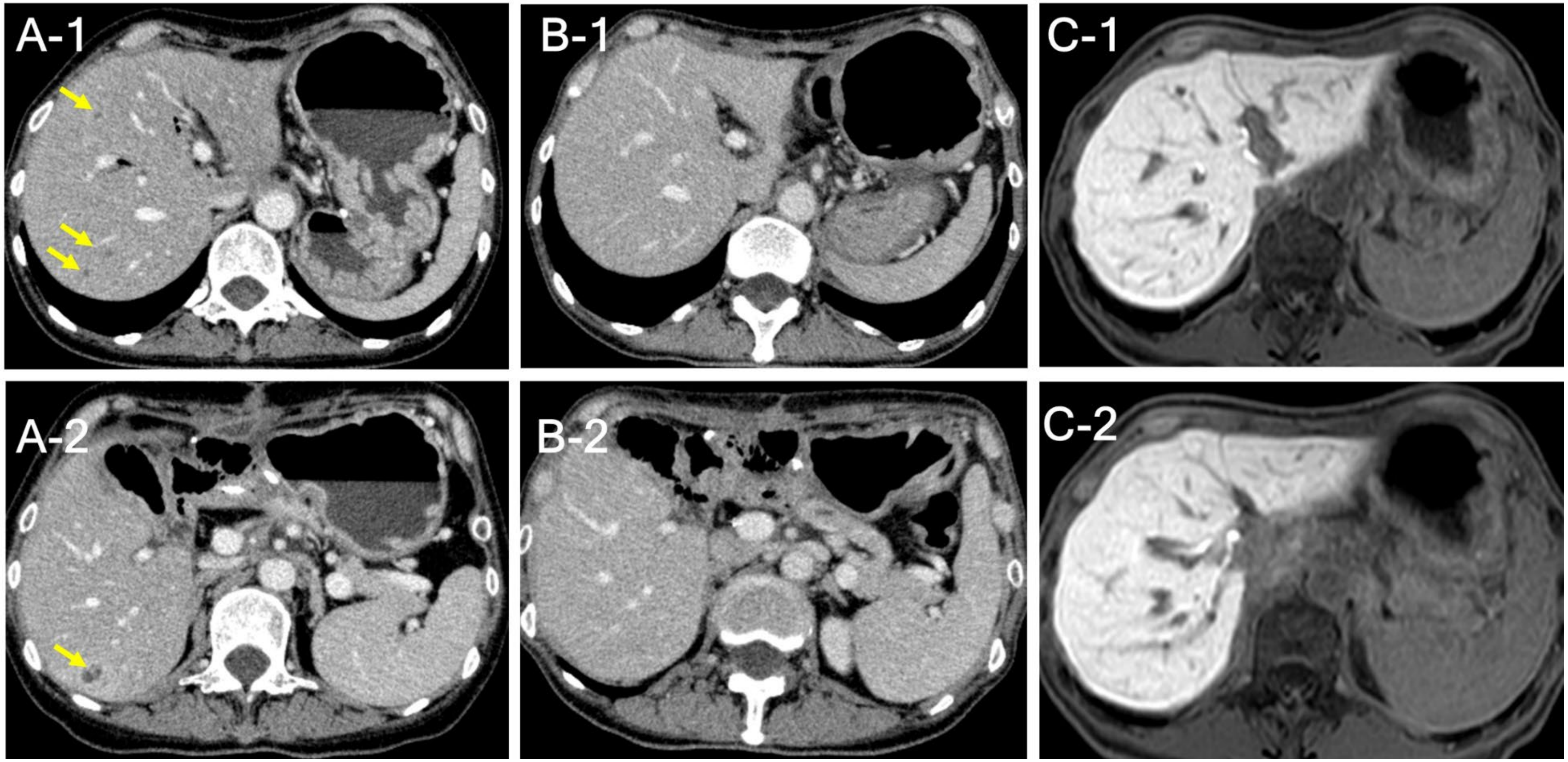

Supplementary Figure 1

Chronological imaging course of transient hepatic lesions after surgery. (A-1,2) CECT performed 2 months postoperatively showed 4 small hepatic lesions (yellow arrow). (B-1,2) At 4 months postoperatively (1 month after initiation of the GCS regimen), all 4 lesions had disappeared on contrast-enhanced CT. (C-1,2) At 11 months postoperatively (6 months after cessation of GCS), no recurrence of these lesions was observed on EOB-MRI.

Abbreviations: CECT, contrast-enhanced computed tomography; GCS: gemcitabine, cisplatin, and S-1; EOB-MRI, gadoxetic acid–enhanced magnetic resonance imaging
